# A Comparative Electromyographic Analysis of Masticatory Muscles Between Skeletal Class II and Skeletal Class I Malocclusion: A Cross-Sectional Study on a Syrian Population

**DOI:** 10.7759/cureus.53960

**Published:** 2024-02-10

**Authors:** Ali A Saker, Mohammad Y Hajeer, Mohamed Youssef

**Affiliations:** 1 Department of Orthodontics, Faculty of Dentistry, University of Damascus, Damascus, SYR

**Keywords:** digastric muscle, muscular activity, perioral muscles, temporal muscle, masseter muscle, electromyography (emg), skeletal class ii malocclusion, skeletal class i malocclusion

## Abstract

Objectives: This study aimed to investigate whether there was a difference in the muscular activity of the masticatory muscles between patients with skeletal Class II and skeletal Class I malocclusion.

Materials and methods: A cross-sectional study was conducted using a sample of 56 selected patients referred to the Department of Orthodontics and Dentofacial Orthodontics, Faculty of Dentistry at the University of Damascus, Damascus, Syria. An electromyographic device measured the myoelectric activity of the perioral muscles on patients in the two created groups: the skeletal Class I malocclusion group (n=28 patients) and the skeletal Class II malocclusion group (n=28 patients).

Results: The study found a similarity in the muscular activity between the right and left sides within the same group, without significant differences between both sides for each muscle (P>0.05). The Class II group had significantly greater activity in the buccinator and digastric muscles than the Class I group (p<0.05). On the other hand, the Class I group had significantly greater activity in the orbicularis and mentalis muscles than the Class II group (P<0.05).

Conclusion: Patients with skeletal Class II malocclusion and skeletal Class I showed differences in muscular activity. The buccinator and digastric muscles were more active in skeletal Class II patients, while orbicularis oris and mentalis were less active. The temporalis and masseter muscles showed similar activity in both groups.

## Introduction

Class II malocclusion is defined as a disorder in the jaw relationship in the sagittal plane, which may be caused by a dimensional or positional disorder of one or both jaws, and it also results from a disorder in the upper or lower dental arch or both [1,2]. As for the pathological mechanism, many studies applied to families and twins have shown that heredity plays an important role in a large percentage of Class II malocclusion cases, especially those of skeletal origin [3,4]. Genetics also plays a role in inhibiting the growth of the lower jaw through intense muscle activity of the orbicularis oris muscle, which leads to palatal inclination of the upper incisors, which in turn leads to lingual inclination of the lower incisors [5].

The impact of muscular forces associated with states of rest and functional performance on tooth positioning remains unclear. It is worth noting that the muscular forces in a functional state are often stronger than those at rest, although they only last for a short period of time. This contrasts with the muscular pressures at rest, which last longer despite their diminished intensity than functional pressures [6].

Ueda et al. studied the electrical activity of the temporalis, masseter, and gastrocnemius muscles in several patients with different facial patterns and found a close correlation between myoelectric activity and facial morphology [7]. Lara-Vianna et al. also found that temporalis and masseter myoelectric activity were related to the growth pattern [8]. On the other hand, Custodio and colleagues found an increase in myoelectric activity in a horizontal growth pattern [9]. Ramsundar et al. found that the amount of protrusion associated with Class II malocclusion did not affect the extent of muscle activity at rest and during chewing [10]. Cha et al. did not find clear differences in the electrical activity of the temporalis and masseter muscles when comparing different growth patterns [11]. Dutra et al. also found no correlation between muscular activity and vertical growth [12]. Alabdullah et al. studied the relationship between facial growth patterns and facial muscle activity and found a significant relationship between facial muscle activity and facial growth patterns [13].

Debate continues over the factors leading to malocclusion, focusing on genetics and environmental elements, especially functional factors. Given that muscular activity associated with the functions of the masticatory system plays a crucial role in the development of the dental-facial complex, the research question was: "Is there a difference in the muscular activity of the masticatory muscles between individuals with skeletal Class II and skeletal Class I malocclusion?"

## Materials and methods

Study design and settings

This study was cross-sectional on patients referred to the Department of Orthodontics at the Faculty of Dentistry, University of Damascus, Damascus, Syria. The myoelectric activity of the perioral muscles was measured at the Department of Biological Sciences at the Faculty of Dentistry, University of Damascus. The Local Research Ethics Committee of the Faculty of Dentistry, University of Damascus, reviewed and approved the protocol of this study (approval no.: UDDS-228-2019HG/SRC1952). This work was funded by the University of Damascus (Ref no: 501100020595).

Sample size estimation

The sample size was calculated using the G-Power 3.1© software (Heinrich-Heine-University, Düsseldorf, Germany). An effect size of 0.36 µv was accepted as the smallest clinically significant difference requiring detection between the two groups regarding myoelectric activity [11]. Applying an independent-samples t-test with a significance level of 0.05 and a power of 85% determined that the appropriate sample size was 56 patients.

Patients recruitment

A clinical examination was conducted for 178 patients from the Department of Orthodontics archives at Damascus University. Of these, 37 Class II and 49 Class I patients have met the inclusion criteria. According to the a priori sample size calculation, 56 patients were selected from the sampling frame and were distributed according to their skeletal occlusal relationships in the sagittal plane into two equal groups as follows: the first group consists of 28 patients with normal skeletal Class I malocclusion and the second group consists of 28 patients who had skeletal Class II malocclusion. All participants or their parents read a written information sheet and signed the consent form. Patients were included in the study based on the following inclusion and exclusion criteria:

Inclusion Criteria

It included the age range between 11 and 14 years, facial height percentage according to Jarabak 60% (±2), a normal overbite (did not exceed one-third of the height of the crowns of the lower incisors and was not less than 2 mm), and no crossbite on one of the back teeth. Patients with Class I skeletal relationship in the midsagittal plane (the ANB angle) = 1-4° for the skeletal Class I group. For the skeletal Class II group, patients with Class II division 1 malocclusion, overjet of more than 5 mm, and Class II skeletal relationship in the midsagittal plane (the ANB angle) >4º.

Exclusion Criteria

The presence of crowding (Little's Irregularity Index ≥ 3), developmental disorders, facial muscle pain, cases of temporomandibular joint disorders, scars or burns on the facial tissues, and previous orthodontic treatment were excluded.

Electromyography

First, the patient was prepared to sit on the dental clinic chair with his head upright and the Frankfurt level parallel to the horizon or the ground. This position was taken as a reference for all patients. The patient’s head was supported on the back of the chair, and based on this, electromyography was performed. The temporalis, masseter, orbicularis, buccinator, mentalis, and digastric muscles were located by palpation [10]. The covered skin was wiped with alcohol, and the forehead was also wiped with alcohol to connect it to the ground electrode (GND), according to the instructions.

The customized gel was placed on the funnel electrodes, which were then attached to the muscle studied using medical adhesive. This ensures the stability of the electrode during planning, with the electrodes of one electrode being 2 cm apart from each other. This procedure was carried out symmetrically on both sides to obtain recordings of the muscles, except for the chin muscle, considered a single muscle.

**Figure 1:**
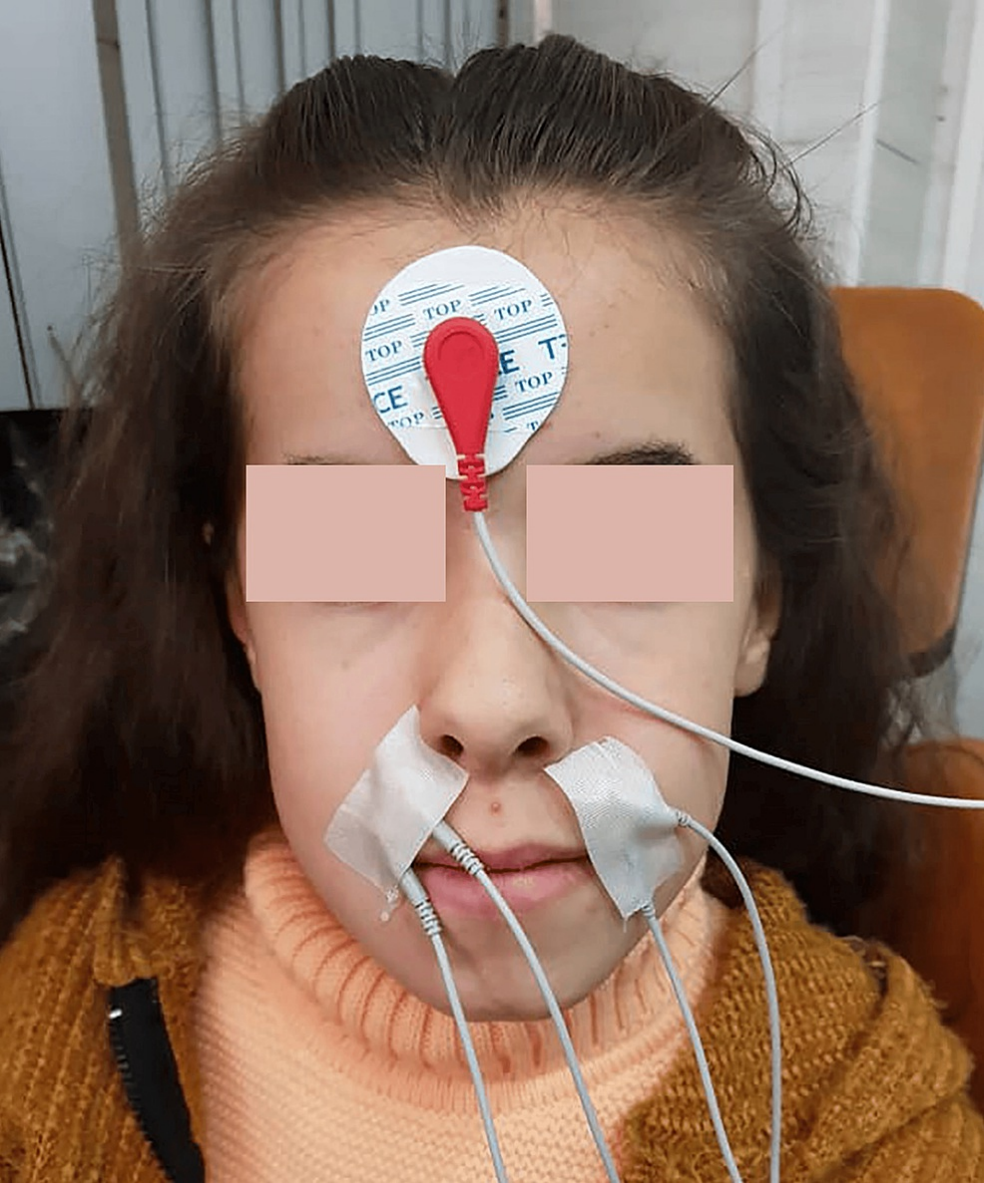
Electromyographic assessment of the orbicularis oris muscle for one of the included patients.

The GND was connected to the forehead (Figure 1), and the program was initiated. The type and location of the planning, the specific muscle, and the type of electrode used were selected, and recording began in each position for five seconds - two seconds before giving the command and two seconds after it, as per the program settings.

**Figure 2:**
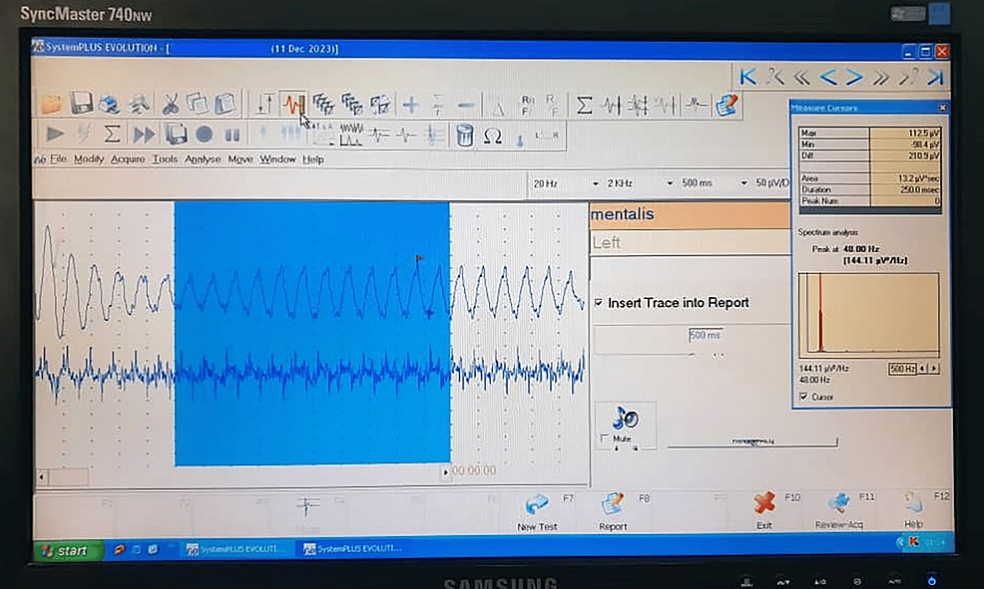
Evaluation of the electromyographic recording using the software.

The command to record was given by pressing the space bar on the keyboard. To ensure no errors in recording or forgetting the planning related to the previous position, planning was done according to a specific sequence and certain conditions in the program. The first track was adopted for the right side and the second for the left side from the program settings. Then, ‘spontaneous’ was chosen for the resting position (Figure 2).

Statistical analysis

Basic statistical tests were conducted using the statistical package SPSS® version 23.0 (IBM SPSS Corp., Armonk, NY, USA). Initially, the distribution of data for all variables used in the study (normal or abnormal) was studied using the Kolmogorov-Smirnov normality test. The independent sample t-test was used to compare the two study groups if the data distribution was normal. In the case of non-normal data distribution, the Mann-Whitney U test was used to detect significant differences between the two groups. The Wilcoxon test was also used to investigate any significant differences between the right and left muscles.

## Results

The results showed similarity in muscle activity (temporalis, masseter, buccinator, digastric, orbicularis oris) on both the right and left sides within the same group without significant differences between both sides for each muscle (P>0.05; Table 1 and Table 2).

**Table 1:** Descriptive statistics of muscle activity on both sides in the Class I malocclusion group and the significance of the differences between the two sides. a: Paired-samples t-test, b: Wilcoxon signed ranks test

		Right (n=28)		Left (n=28)		Mean Difference	95% Confidence Interval		P-value
		Mean±SD	Median	Mean±SD	Median		Lower	Upper	
Temporalis muscle	MIN	-43.33 ± 25.08	-37.8	-40.76 ± 22.07	-36.6	-2.57	-6.67	1.54	0.198^b^
	MAX	43.5 ± 21.3	38.35	44.46 ± 22.28	37.5	-0.96	-6.36	4.43	0.311^b^
	OVERALL	86.83 ± 44.61	76.95	85.23 ± 42.96	75.8	1.60	-5.34	8.55	0.767^b^
Masseter muscle	MIN	-38.67 ± 8.68	-39.75	-38.08 ± 9.21	-35.65	-0.59	-3.45	2.26	0.673^a^
	MAX	42.76 ± 11.71	43.2	40.31 ± 10.23	39.45	2.45	-2.79	7.69	0.346^a^
	OVERALL	81.43 ± 18.04	82.6	78.38 ± 16.28	74.6	3.04	-3.60	9.68	0.355^a^
Buccinator muscle	MIN	-32.85 ± 11.28	-32.2	-33.32 ± 10.92	-35.9	0.47	-3.95	4.88	0.829^a^
	MAX	35.23 ± 10.45	33.15	35.85 ± 9.34	33.75	-0.63	-4.53	3.27	0.743^a^
	OVERALL	68.08 ± 18.73	67.6	69.18 ± 17.48	73.6	-1.10	-9.01	6.81	0.778^a^
Digastric muscle	MIN	-43.73 ± 14.94	-39.1	-39.82 ± 12.21	-37.85	-3.91	-10.12	2.30	0.269^b^
	MAX	42.02 ± 16.52	36.8	40.59 ± 13.91	38.3	1.43	-5.11	7.97	0.597^b^
	OVERALL	85.75 ± 30.82	73.75	80.41 ± 25.88	74.2	5.34	-7.07	17.75	0.767^b^
Orbicularis oris muscle	MIN	-36.98 ± 9.46	-35.5	-34.62 ± 9.73	-34.1	-2.36	-6.11	1.39	0.207^a^
	MAX	37.07 ± 9.08	35.5	37.85 ± 9.11	35.9	-0.78	-4.76	3.20	0.642^b^
	OVERALL	74.05 ± 17.66	72.4	72.46 ± 15.18	72.55	1.58	-5.07	8.23	0.630^a^

**Table 2:** Descriptive statistics of muscle activity on both sides in the class II malocclusion group and the significance of the differences between the two sides. a: Paired samples t-test, b: Wilcoxon signed ranks test

		Right (n=28)		Left (n=28)		Mean Difference	95% Confidence Interval		P-value
		Mean±SD	Median	Mean±SD	Median		Lower	Upper	
Temporalis muscle	MIN	-40.13 ± 17.84	-38	-41.58 ± 25.57	-37.35	1.45	-9.49	12.39	0.946^b^
	MAX	41.79 ± 18.74	38	37.62 ± 15.92	33.25	4.16	-2.60	10.93	0.724^b^
	OVERALL	81.91 ± 36.31	76.65	79.2 ± 37.93	69.4	2.71	-13.83	19.26	0.739^a^
Masseter muscle	MIN	-36.79 ± 11.15	-34.4	-38.22 ± 17.59	-34.6	1.43	-6.58	9.43	0.873^b^
	MAX	43.36 ± 19.83	35.15	41.58 ± 20.13	33.6	1.78	-7.59	11.16	0.891^b^
	OVERALL	80.15 ± 27.2	70.65	79.79 ± 35.88	69.25	0.36	-15.25	15.96	0.716^b^
Buccinator muscle	MIN	-48.97 ± 16.58	-44.65	-45.71 ± 13.46	-42.8	-3.26	-9.40	2.88	0.955^b^
	MAX	49.63 ± 14.4	48.15	45.73 ± 12.31	43.95	3.90	-1.49	9.29	0.759^b^
	OVERALL	98.6 ± 30.32	90.15	91.44 ± 22.59	89.05	7.16	-3.45	17.78	0.973^b^
Digastric muscle	MIN	-51.54 ± 17.14	-48.75	-50.31 ± 13.37	-50.3	-1.23	-7.51	5.05	0.691^a^
	MAX	52.01 ± 19.25	48.6	49.76 ± 13.02	49.25	2.25	-4.67	9.16	0.511^a^
	OVERALL	103.55 ± 32.61	99.35	100.1 ± 25.69	98.65	3.48	-7.83	14.78	0.533^a^
Orbicularis oris muscle	MIN	-31.27 ± 7.01	-30.8	-28.56 ± 9.32	-27.9	-2.71	-6.99	1.56	0.204^a^
	MAX	32.46 ± 8.06	32.4	32.83 ± 11.15	29.9	-0.37	-5.37	4.62	0.876^b^
	OVERALL	63.73 ± 12.28	63.6	61.39 ± 14.9	59.15	2.34	-3.47	8.16	0.554^b^

The mean activity of the buccinator muscle was significantly greater in the Class II group (95.02 µv ± 22.96 µv) compared to the Class I group (68.63µv ± 14.97 µv), with a significant difference between the two groups (P<0.001). Similarly, the mean activity of the digastric muscle was also significantly greater in the Class II group (101.81µv ± 25.48 µv) compared to the Class I group (83.08µv ± 23.53µv), with a significant difference (P=0.001).

On the contrary, the mean activity of the orbicularis muscle was significantly greater in the Class I group (73.26 µv ± 14.06µv) compared with the Class II group (62.56µv ± 11.41µv, P=0.003). Similarly, the mean activity of mentalis muscle was also significantly greater in the Class I group (92.01µv ± 27.02µv) compared with the Class II group (70.5 µv ± 17.51µv, P=0.001; Table 3).

**Table 3:** Descriptive statistics of muscle effectiveness in the two malocclusion groups and the significance of the difference between the two groups * Significant at the 0.05 level a: Independent samples test, b: Mann-Whitney test.

		Class I group (n=28)		Class II group (n=28)		Mean Difference	95% Confidence Interval		P-value
		Mean±SD	Median	Mean±SD	Median		Lower	Upper	
Temporalis muscle	MIN	-42.04 ± 23.02	-35.9	-40.85 ± 16.95	-38.55	-1.19	-12.0	9.64	0.555^b^
	MAX	43.98 ± 20.66	38.575	39.7 ± 15.04	34.725	4.28	-5.40	13.96	0.417^b^
	OVERALL	86.03 ± 42.87	75.825	80.56 ± 30.38	75.675	5.47	-14.44	25.38	0.967^b^
Masseter muscle	MIN	-38.37 ± 8.15	-37.875	-37.51 ± 10.5	-35.075	-0.87	-5.9	4.17	0.326^b^
	MAX	41.53 ± 8.67	40.175	42.47 ± 15.91	36.625	-0.93	-7.84	5.98	0.528^b^
	OVERALL	79.9 ± 14.9	79.825	79.97 ± 24.67	72.35	-0.07	-11.04	10.91	0.367^b^
Buccinator muscle	MIN	-33.09 ± 9.53	-34.15	-47.34 ± 12.86	-45.7	14.25	8.19	20.31	<0.001^a^*
	MAX	35.54 ± 8.55	33.125	47.68 ± 11.45	46.125	-12.14	-17.55	-6.73	<0.001^b^*
	OVERALL	68.63 ± 14.97	66.8	95.02 ± 22.96	91.6	-26.39	-36.78	-16.00	<0.001^b^*
Digastric muscle	MIN	-41.78 ± 11.05	-38.65	-50.92 ± 13.06	-48.35	9.15	2.67	15.97	0.003^b^*
	MAX	41.31 ± 12.73	37.675	50.88 ± 13.8	48.8	-9.58	-16.69	-2.46	0.002^b^*
	OVERALL	83.08 ± 23.53	75.3	101.81 ± 25.48	99.05	-18.73	-31.87	-5.59	0.001^b^*
Orbicularis oris muscle	MIN	-35.8 ± 8.29	-34.45	-29.91 ± 6.13	-29.875	-5.88	-9.79	-1.98	0.004^a^*
	MAX	37.46 ± 7.51	37.175	32.64 ± 7.29	32.825	4.81	0.85	8.78	0.018^a^*
	OVERALL	73.26 ± 14.06	72.6	62.56 ± 11.41	59.95	10.70	3.84	17.56	0.003^a^*
Mentalis muscle	MIN	-45.68 ± 15.76	-43.3	-34.01 ± 8.55	-33.6	-11.66	-18.51	-4.83	0.001^a^*
	MAX	46.33 ± 12.92	42.05	36.49 ± 10.12	34.4	9.84	3.62	16.06	0.001^b^*
	OVERALL	92.01 ± 27.02	85.55	70.5 ± 17.51	67.2	21.50	9.26	33.75	0.001^b^*

## Discussion

The orthodontist must know the mechanism of the facial muscular template and its impact on the growth and development of the craniofacial complex. Therefore, this research aimed to compare the activity of the masticatory and perioral muscles in subjects with skeletal Class II malocclusion and skeletal Class I malocclusion in the sagittal plane.

The increased interest in electromyography in orthodontics is due to the need for reliable and effective treatment of malocclusion. Awareness of the causes of malocclusion emphasizes the impact of function on form and justifies the goals of functional orthodontic treatment, which aims to improve the balance of the masticatory muscles and promote the correct development of the facial skeleton. Electromyography provides an objective, non-invasive assessment of the masticatory muscles [10].

The masseter and temporalis muscles are considered important muscles during mastication because they work to raise the lower jaw and move it laterally and posteriorly [10]. There was no significant difference between the right and left sides. This result agreed with Ramsundar et al., Ardani et al., and Cha et al. [10,11,14]. This can be explained by the lack of a unilateral chewing habit in most patients in this study. Bakke stated that the presence of a unilateral chewing habit can lead to an increase in muscle size on the chewing side and, thus, an increase in muscle activity for the muscles on this side [15].

No significant statistical differences were observed between the Class I and Class II patients in the activity of the masseter and temporalis muscles in the resting position of the mandible. This result differed from that of Ardani et al., who found greater muscle activity in the skeletal Class II with the masseter and temporalis muscles compared to the skeletal Class I [14]. The difference can be attributed to the difference in age range, as in his study, the age of the patients was between 18 and 22 years, while in the current study, the age of patients was between 11 and 14 years.

The mean buccinator muscle activity was significantly greater in Class II patients, and this agrees with the results of Stavridi and Ahlgren’s study [16], where this excessive activity of the buccinator muscle is attributed to its causative role in the development of narrow dental arches [17]. The mean muscle activity of the digastric muscle was significantly greater in Class II compared to Class I, indicating increased function and use that may affect the growth of the lower jaw in the sagittal direction. These findings agreed with those of Petrović et al. [18].

Strengths and limitations of the current study

This study is the first to compare perioral muscle activity between patients with Class II and Class I malocclusions. However, there are some limitations. For instance, muscle activity was evaluated only once, specifically in the resting position. Future research could be conducted to determine muscular activity in other positions or after the correction of skeletal malocclusion. Additionally, the relatively young age of the sample is a limitation of this study, highlighting the need for subsequent studies on muscle activity in older age groups with different classes of skeletal malocclusion.

## Conclusions

The muscular activity was not completely similar between patients with skeletal Class II malocclusion and skeletal Class I. The muscular activity was similar for the temporalis and masseter muscles only, while there were significant differences in the activity of the buccinator, mentis, orbicularis oris, and digastric muscles. Skeletal Class II patients had significantly greater muscle activity in the buccinator and digastric muscles than skeletal Class I patients, while their muscle activity in the orbicularis oris and mentalis was lower.
